# Selected Properties of a TPS/PA12 Composite Material Produced in a Two-Stage Method

**DOI:** 10.3390/polym17111517

**Published:** 2025-05-29

**Authors:** Ewa Tomaszewska-Ciosk, Ewa Zdybel, Małgorzata Kapelko-Żeberska, Beata Anwajler

**Affiliations:** 1 Department of Food Storage and Technology, Wroclaw University of Environmental and Life Sciences, 25 Norwida Street, 50-375 Wroclaw, Poland; ewa.tomaszewska-ciosk@upwr.edu.pl (E.T.-C.); malgorzata.kapelko-zeberska@upwr.edu.pl (M.K.-Ż.); 2 Department of Energy Conversion Engineering, Faculty of Mechanical and Power Engineering, Wroclaw University of Science and Technology, 27 Wybrzeze Wyspianskiego Street, 50-370 Wroclaw, Poland

**Keywords:** biodegradable composite material, polyamide, thermoplastic starch

## Abstract

The world economy is struggling with the increasing pollution of the natural environment with non-biodegradable synthetic polymers produced from petroleum products. This fact has prompted research on the use of natural renewable polymers. Starch is one of the polymers that has already been used as an additive to synthetic polymers; however, its use is associated with a problem arising from the incompatibility of hydrophilic starch with hydrophobic synthetic polymers. For these reasons, other authors have not used more than 20% of the starch component in synthetic materials. In this work, a research hypothesis was put forward that the starch content can be increased in the polymer material. Pre-extrusion was used before the final material molding process. Pre-extrusion improved the phase dispersion of the synthetic polymer blended with starch. To produce the molds, the polyamide and starch blends were subjected to the processes of extrusion, milling, and pressing. The molded samples containing polyamide and starch were obtained with a starch component content of 50, 70, and 90%. The obtained homogeneous material was determined in terms of its water resistance and mechanical properties. The test results showed that increasing the starch content in the produced material, increased its susceptibility to water, and worsened its strength properties. However, these negative effects were not as large as expected, and in some cases were even statistically insignificant. The addition of 70% of the starch component allowed for the production of a composite material with satisfactory mechanical properties.

## 1. Introduction

Due to the increasing environmental pollution with non-biodegradable synthetic polymers made of petroleum-derived products, an urgent need has emerged to reduce their production. This can be achieved by creating safer synthetic materials [1,2] or replacing them with biodegradable materials [3]. For years, this problem has been addressed by research into the use of natural renewable polymers (cellulose [4,5], chitosan [6,7,8], starch [3,9,10]), and the biodegradable synthetic polymers (poly(lactic acid) (PLA) [11,12,13], polycaprolactone (PCL) [14], polyhydroxybutyrate-valerate (PHBV) [15], polyhydroxyalkanoate (PHA) [16], and polyesteramide [17]). Currently, the market leaders among biodegradable polymers are those based on starch [3], which is a cheap, easily renewable, non-toxic, and rapidly biodegradable material; this makes it a very attractive alternative to other biopolymers. An additional advantage of starch is its chemical structure, i.e., it consists of glucose molecules linked with alpha 1-4 and alpha 1-6 glycosidic bonds, containing as many as three hydroxyl groups [9]. Such a structure enables easy modification via chemical methods using a variety of compounds. This also allows for the production of a practically unlimited number of modified preparations exhibiting various properties. Starch can also be easily modified via physical and enzymatic methods, which further multiplies the number of products that can be developed [3,9]. The production of thermoplastic starch (TPS) is the most frequently deployed starch modification method for the production of biopolymers. It entails the chemical, thermal, and/or mechanical modification of starch, which results in the swelling of its globules and destruction of its crystalline structure, followed by gelatinization and plasticization [3]. Due to the large number of hydroxyl groups, TPS is strongly hydrophilic, which is associated with its biodegradability, but at the same time narrows the possibilities for practical applications [18]. In order to eliminate the undesirable properties of starch and other natural polymers, research has been underway for many years to identify the possibility of using mixtures of these with synthetic polymers [3,4,5]. Starches used as fillers in traditional plastics improves the cost-effectiveness of bioplastics production. Numerous studies have confirmed that such plastics meet the expectations of having good functional properties (which are ensured by the synthetic polymers) and an increased biodegradability resulting from the presence of natural polymers [8,9,11,19]. It should be noted, however, that the starch content of these mixtures is limited to a certain range, above which the functional properties of such plastics deteriorate drastically [20]. Furthermore, it has been observed that the increased biodegradability of these materials does not eliminate the problem of microplastics. Of course, less microplastics are produced than from conventional petroleum-derived polymers, but within a shorter time [20]. In addition, a problem from the immiscibility of hydrophilic starch with hydrophobic synthetic polymers has emerged during the production of materials comprising synthetic polymers and natural fillers [21]. In order to tackle this problem, many authors have undertaken attempts to investigate the possibility of using various substances that act as plasticizers. These have significantly reduced the size of the dispersed phase particles and at the same time improved their interphase adhesion. These authors have observed an improvement in the viscosity and elasticity of TPS, and consequently in its morphology and functional properties, by using such plasticizers as water [21], glycerol [22], citric acid [21], urea [21], and sorbitol [4,5]. Increasing the content of plasticizers in plasticized starch by adding polyethylene, has been shown to increase the size of well-dispersed starch regions within the polyethylene matrix [21,23,24]. Other authors studying TPS/PE composite materials with the addition of maleic anhydride, have also observed an improvement in the morphology of the modified material due to a reduction in the size of the dispersed phase particles (TPS) [23]. However, in the case of the composite material made from thermoplastic starch and polypropylene (TPS/PP), a significant deterioration of its mechanical properties was noted, despite very good dispersion of the starch phase, including a reduction in tensile strength by more than half, and elongation at break by up to 200% [23,25]. Most substances that effectively improve the dispersion of composite material phases tend to be toxic or exert adverse effects on the natural environment during their production or biodegradation. For this reason, other methods are being intensively sought that will ensure phase continuity in composite materials. Another successful attempt to reduce the polarity at the composite material phase mixing interface, has been to use compounds with a more polar nature, e.g., polyurethanes (PU) [26]. This TPS/PU composite material is characterized as having good dispersion of its components due to hydrogen bonding interactions between the PU and TPS, which results in the improvement in both the mechanical properties and water resistance of the resulting composite materials. Polyamide PA is another compound with a more polar nature compared with other synthetic polymers [23]. Despite this, it has not attracted much interest, compared with other polar polymers, as a component of starch-based composite materials. In most cases, polyamides are non-biodegradable derivatives of petroleum with a very high melting point (exceeding 250 °C) [27]. Such a high melting point limits their use in starch mixtures due to the very intensive thermal degradation of starch that occurs under these conditions. The polyamide, PA11, stands out from other polyamides not only due to its much lower melting point (180 °C), but also because of the fact that it is synthesized from easily renewable castor oil and has a long hydrophobic aliphatic chain, which ensures its high water resistance [22]; therefore, it combines very important commercial properties that makes it a recommended component of composite materials that are made with thermoplastic starch, i.e., greater polarity (required for good adhesion to the starch) as well as hydrophobicity and high tensile strength (required to improve the greater friability associated with the starch components of such a composite material).

Another method that could also eliminate the problem of polymer immiscibility at the interface, involves pre-extrusion prior to the actual process of plastics formation from the mixture [12]. Such an operation might not only improve the dispersion of the particle phase in the material, but might also eliminate the problem of material delamination. In order to reduce the amount of microplastics, it is important to increase the content of the biodegradable component (e.g., starch) in the mixture. Unfortunately, previous studies have indicated that such a procedure significantly reduces the mechanical strength of most plastics and increases their susceptibility to the effects of water. The PA11 polyamide is also one of the few materials whose mechanical properties are not compromised, even with a high contribution of the TPS component [22]. Unfortunately, its high price makes it cost-ineffective for use in commercial applications. However, it is likely that a similar effect can be achieved by using its cheaper version, i.e., PA12, which is also characterized as having a lower melting point that does not induce starch degradation. In this context, it seems important to scrutinize the properties of a TPS/PA12 composite material, by harnessing an additional extrusion process prior to the actual molding of the material (for example during pressing or 3D printing). It is probable that such a procedure can lead to better dispersion of both polymers and, thus, result in improved functional properties of the produced composite material. A research hypothesis was advanced for the present work that the starch content can be increased in a polymeric material via pre-extrusion prior to the actual process of material molding using a synthetic polymer/starch mixture, and that this may improve phase dispersion. Therefore, the aim of this study was to determine the effect of TPS content and a two-stage production process on the properties of molded specimens produced from a TPS/PA12 composite material.

## 2. Materials and Methods

### 2.1. Preparation of Samples

**Figure 1:**
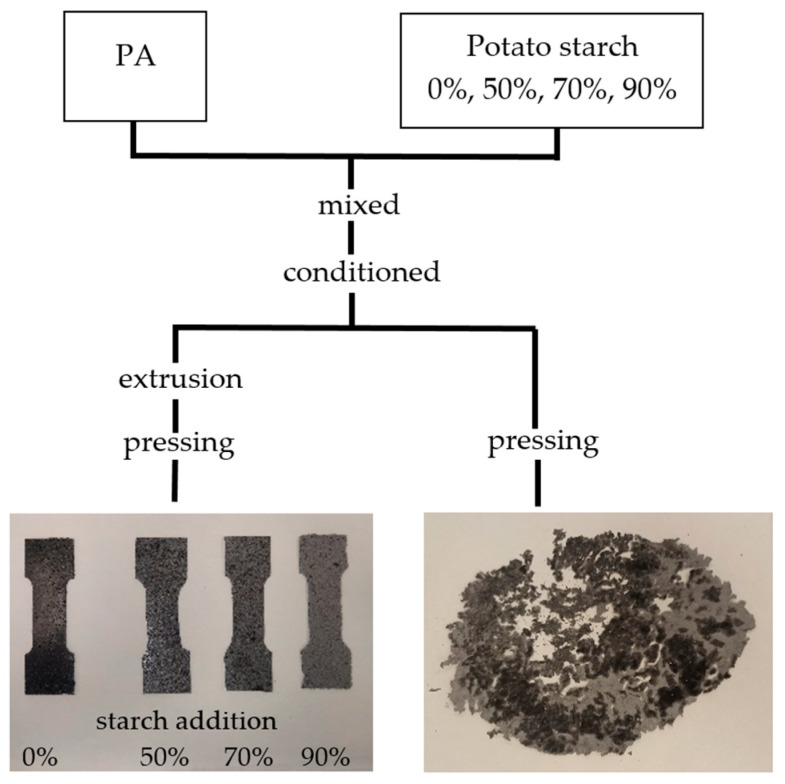
Schematic of the preparation of samples.

Potato starch (PEPEES Łomża 2024) with a moisture content of 25% was added to a PA12 polyamide (Sanit 2023) in the amounts of 0%, 50%, 70%, and 90% of starch dry matter. Next, the samples were thoroughly mixed and conditioned in polyethylene bags at a temperature of 25 °C for 24 h to equalize the moisture content. Each of the mixtures was divided into two portions; one of which was subjected to pre-extrusion followed by pressing, and the other only to pressing, as shown in Figure 1.

### 2.2. Extrusion Process

Extrusion was performed in a Brabender 20DN single-screw extruder, at 90 °C in the first section, 115 °C in the second section, and 140 °C in the third section. A round nozzle with a diameter of 2 mm, and a screw with a compression ratio of 2:1 rotating at a speed of 100 rpm, were used in the process. The extruded material was fed at a speed of ca. 200 rpm adjusted so that the engine load did not exceed 7A. After the extrusion process had been completed, all the samples were conditioned at a temperature of 25 °C for 24 h and then ground using a laboratory mill to a fraction size of 0.1 mm. Afterwards, distilled water was added to each sample to compensate for the water lost during extrusion. All the samples were placed in polyethylene bags and conditioned again at a temperature of 25 °C for 24 h to equalize their moisture content. All the applied extrusion and conditioning parameters were selected based on previous studies and the authors’ reports [28].

### 2.3. Pressing Process

Pressing was performed using a laboratory press (own design). A 20-g bulk of the TPS/PA mixture was transferred into the press with a plate (20 cm in diameter) between two Teflon sheets heated to 180 °C. The mixtures were left therein, without pressure, for 5 min to liquefy the bulk, and then the press pistons were clamped until a pressure of 200 kg/cm^3^ was obtained. The samples were left under these conditions for 2 min. After cooling the press to 50 °C, the samples were removed from the mold. Next, the sheets were cooled to room temperature and conditioned at 25 °C for 24 h. All the applied pressing and conditioning parameters were selected based on previous studies and the authors’ reports [3]. Paddle-shaped specimens were cut out from the formed sheets with dimensions compliant with the PN EN ISO 527-2:2012 Standard [28].

### 2.4. Analyses

#### 2.4.1. Mechanical Properties

The mechanical properties of the molded specimens were measured using an Instron 5544 tensile tester (Norwood, MA, USA) with a breaking attachment, a measuring head enabling measurements in the range of up to 100 N and moving at a speed of 1 mm/s (the Instron 5544 tensile tester was calibrated using samples made of pure polyethylene.). The pressed specimens were placed in the breaking attachment, and the values of the force acting on them were recorded at a frequency of 16.7 s^−1^ [28]. The analyses were carried out until the pressed molds broke. The plotted correlations between the breaking force and time allowed for determining the minimum force (F) required to break the molded specimens, and for calculating the tensile strength, the minimum work required to break, and the amount of deformation (the percentage elongation at break). The data are presented as the mean of 30 measurements [29].

#### 2.4.2. Resistance to Water

The water resistance of the molded specimens was determined using whole samples cut out as specified in Section 2.4.1. Once weighed, the molded specimens were placed in a flask with distilled water and shaken in a water bath with a temperature of 30 or 80 °C for 30 min, at a speed of 80 rpm. Afterwards, they were separated from the water, weighed, and dried at 105 °C for 48 h. The dried samples were weighed again. The weights of the molded specimens measured before and after soaking in water enabled computation of material solubility at temperatures of 30 and 80 °C, as well as the theoretical water absorption and practical water absorption (considering the material’s solubility) at these temperatures [30]. The applied parameters for determining the water resistance were selected based on previous studies and the authors’ reports [31]. The data are presented as the mean of five measurements.

The following formulas were used for calculations:

(1) $$S=\frac{m_{0}-m_{D}}{m_{0}}\cdot 100\%$$

(2) $$W_{T}=\frac{m_{W}-m_{0}}{m_{0}}$$

(3) $$W_{P}=\frac{m_{W}-m_{0}}{m_{D}}$$

S—solubility [%]

$W_{T}—$ theoretical water absorption [g/g]

$W_{P}—$ practical water absorption [g/g]

$m_{0}—$ initial sample weight [g]

$m_{D}—$ weight of the dried sample [g]

$m_{W}—$ weight of the wet sample [g]

### 2.5. Statistical Analysis

The study results were statistically analyzed using Statistica 12.0 StatSoft (StatSoft, Cracow, Poland). They were subjected to one-way analysis of variance and then analyzed using Duncan’s test to compare the mean values (homogenous groups and LSD values were determined). Any differences were considered statistically significant at *p* < 0.05. The averaged results from all the replicates are presented in the table and in the figures, where the calculated homogeneous groups are marked in lower case letter; additionally, error bars are shown in the figures.

## 3. Discussion of Results

During an attempt to produce polyamide composite materials with at least 50% starch content via conventional extrusion on a press, the products that were obtained were so brittle that they could not be removed from the press in one piece (therefore, they were eliminated from further analyses). A durable product was obtained only when the material molding in the press was preceded by its pre-extrusion, which resulted in an earlier initial dispersion of the starch occurring under the conditions of mixing both polymers in a semi-liquid phase. A durable product from the two-stage process was obtained despite the use of a temperature of only 140 °C during the extrusion process, which is even lower than that used in the pressing process (with identical pressures during both processes). This effect can be explained by the shear forces acting during the extrusion process, which significantly improve the dispersion of the material during its movement inside the barrel.

### 3.1. Water Resistance

In order to determine the material’s resistance to water, the produced specimens were analyzed for their solubility in water at 30 °C and 80 °C, as well as their theoretical and practical water absorption at these temperatures. The tested material was characterized as having partial solubility in water, which had an impact on their water absorption value. In order to take this fact into account, the water absorption corrected for the solubility value was calculated. Because the corrected value is more important within industrial practices, it was defined as “practical water absorption”.

**Figure 2:**
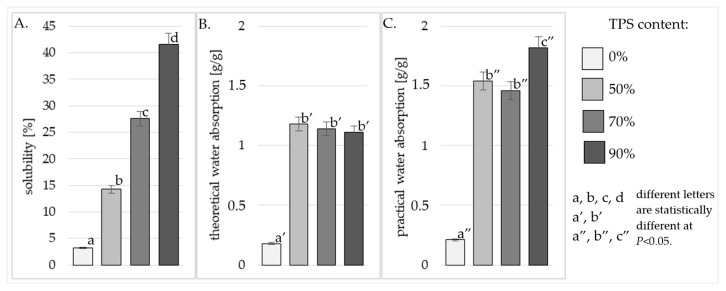
Solubility in water (**A**), theoretical water absorption (**B**), and practical water absorption (**C**) at 80 °C of composite material with different TPS content.

**Figure 3:**
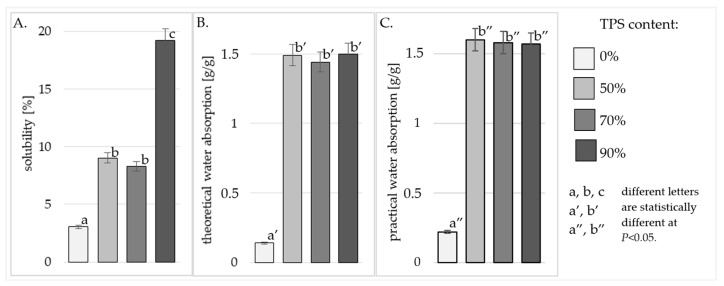
Solubility in water (**A**), theoretical water absorption (**B**), and practical water absorption (**C**) at 30 °C of composite material with different TPS content.

Analyzing the data in Figure 2A, it was found that the solubility of the produced specimens analyzed at 80 °C increased with an increase in the starch content in the material. At this temperature, 41.57% of the specimen containing 90% dissolved starch, whereas the solubility of the molded specimen with 70% starch content was significantly (statistically) lower and reached 27.60%. Among the molded specimens produced with the starch component, statistically the lowest result was obtained for those containing equal amounts of starch and powder in their formula (14.24%). In the water with a temperature of 30 °C, the specimens with 90% starch content were also dissolved to the greatest extent, and their average solubility reached 19.26% (Figure 3A.). However, no statistically significant differences were found in the solubility of the specimens with less than 90% starch content. In this case, their solubility was more than twice lower compared with the molded specimens produced with the highest share of starch; this reached 8.30% (in those with 70% starch) and 9.04% (in those with 50% starch). The solubility of polymers in water is a feature that had the greatest impact on the susceptibility of the material to enzymes and its biodegradability. Other authors have shown in their studies, a positive correlation between solubility and water absorption and the degree of biodegradation of plastics [32]. An increase in solubility causes the porosity of the material to increase. The presence of pores facilitates biodegradation by increasing the surface area of enzyme activity. Additionally, an increase in water absorption by the material, increases water activity and thus enables an increased development of microorganisms and the amylolytic enzymes that they produce [32]. TPS is a hydrophilic polymer due to the very large number of its hydroxyl groups; therefore, the larger share of the starch component in the starch/polyamide mixture results in the production of a composite material with a greater affinity for water. Thus, it seems obvious that the solubility of various composite materials containing TPS increased with its increasing content in the mixture [20]. The solubility of the biocomposites produced in this study was definitely lower than might be expected, based on the TPS component increases in the biocomposite blends. Nazrin et al. in their work, also analyzed the solubility of composites with a high content of TPS (20–80%) and determined a 68% higher solubility at the same dissolution temperature compared with the results obtained in the present study [33]. This effect is probably due to a high degree of homogeneity of both polymers in the composite. Other authors have noted a tangible solubility increase for samples with an insufficient dispersion of the TPS within the polymer matrix (manifested by agglomerates of starch granules visible in the SEM image) [34].

When describing the water absorption capacity of TPS/PA composite materials, it should be remembered that the starch component of this polymer is highly soluble in water, especially at higher temperatures. This observation directly addresses material hydrophilicity and temperature-dependent solubility; this aligns with the discussion about superhydrophobic coatings (STA@TiO_2_/PU), which are designed to mitigate water-induced corrosion in Al-Li alloys. Both contexts emphasize controlling water interaction to enhance material durability [35]. For this reason, in the present study, we not only investigated the effect of the solvent temperatures on water absorption in general, but also determined two types of water absorption: theoretical (Figure 2B and Figure 3B) (describing the volume of water absorbed by the entire dry matter of the TPS/PA mixture contained in the product), and practical (Figure 2C and Figure 3C) (taking into account the volume of water absorbed only by the TPS/PA component of the mixture not dissolved in water). The theoretical water absorption of the tested composite materials ranged from 1.11 to 1.50 g water/g mixture. The statistical analysis showed no significant differences in the theoretical water absorption between all the tested TPS/PA composite materials, at both the analyzed measurement temperatures (Figure 2B and Figure 3B). Similar results were also achieved in the case of the practical water absorption of the composite materials determined at 30 °C, i.e., no statistically significant differences were shown in the value of this parameter depending on the starch component content in the composite materials (Figure 3C). Only the practical water absorption of the composite materials analyzed at 80 °C was dependent on the starch content of the composite material mixture. At this temperature, the composite material with the highest share of TPS (90% TPS) absorbed the highest amount of water (1.82 [g/g]) (Figure 2C). In contrast, the composite materials with 70% and 50% starch content did not differ significantly (statistically) in their water absorption capacity, which was 1.46 [g/g] and 1.54 [g/g], respectively (Figure 2C). Among the polar polymers, polyamides have rarely been studied as components of starch-based composite materials, because most polyamides have a very high melting point, at which point starch undergoes thermal degradation. In the few available works addressing the analysis of the water resistance and water absorption of TPS/PA composite materials, the authors analyzed the volume of water absorbed during contact with air of a specific humidity (water in the form of an aerosol), rather than water in a liquid form. The kinetics of water absorption in such conditions also proceeds in an exponential function, but at a much slower rate. Determination of the equilibrium value of water absorption in TPS/PA composite materials in humid air takes up to several days [36]. Additionally, when studying water absorption from the air, it is not possible to consider the starch solubility process which occurs in aqueous solutions. As shown in Figure 3, analyses of solubility cannot be omitted, because it has a very strong impact on the practical water absorption values that were obtained. Ladnreau noted in their work that composite materials rich in TPS not only achieve higher water absorption values, but also reach the equilibrium point of moisture absorption faster than those with a high PA content. They linked this fact with water solubility, which is higher in the starch phase than in the PA phase. The lack of statistically significant differences in water absorption between the composite materials produced with 50% and 70% TPS can, therefore, be attributed to the very good dispersion of both components within this composite material. This resulted in a more continuous occurrence of the TPS phase in this starch-rich material, which also contributed to the reduced solubility of these composites. Such an effect has not been achieved by other authors in their works in which starch was formed during a single-stage pressing or extrusion. Water absorption and solubility were calculated not for the entire mass of the composite material mixture, but for the content of the starch component performed in these works. This produced results similar to those obtained for crude TPS starch [8]. Thus, it is clear that it is impossible to obtain a good-quality blend of the hydrophobic PA11 and the hydrophilic starch, using a single-stage extrusion process of the TPS/PA11 composite material. For this reason, in Landreau’s work, PA11 did not affect the water absorption of the TPS, whereas the reduction in water absorption by composite materials rich in PA11 was only due to the reduced content of the highly water-soluble starch component of these polymers [36].

### 3.2. Mechanical Properties

Another difficulty posed by the use of TPS in a mixture with PA is deterioration of its mechanical properties, as evidenced in many works [19,21,37,38]. Table 1 presents the effect of the content of the TPS component in the composite material on selected mechanical properties determined during elongation of the molded specimens. As the data from Table 1 indicate, values of their mechanical properties decreased inversely proportional to the starch content. The highest minimum force was determined for the specimens containing 50% and 70% TPS, which did not differ significantly (statistically) between each other. Their minimum breaking forces were 68.37 [mN] and 63.39 [mN], respectively, and were lower than the force obtained for pure PA by only about 12%. The lowest minimum force was noted in the case of the composite material produced with 90% TPS (53.59 [mN]). Similar results were also obtained in the case of tensile strength and the work necessary to break the molded specimen. In the case of these values, no differences were observed in the samples containing up to 70% TPS. In turn, increasing the starch component content to 90% caused a decrease in these values, especially in the case of the work required to break the molded specimens, which decreased to 25% of the work required to break the samples with 50% TPS (Table 1). The deterioration of mechanical properties is related to the difficulties in obtaining an appropriately dispersed hydrophilic phase of TPS and other mostly hydrophobic polymers, because of a lack of adhesion between these polymers [21,23]. This statement highlights the relationship between material microstructure (phase dispersion) and mechanical performance, paralleling the analysis of gradient microstructures in induction-quenched steel and their impact on crack propagation mechanisms. Both studies link structural homogeneity to enhanced mechanical properties [39]. The solution to this problem may be to produce composite materials using polymers with a smaller difference in polarity, e.g., poly(esteramide) and polyurethane [22,24], or a plasticizer that can reduce the tension at the interface of the polymers used [26]. In these cases, an improvement in dispersion was achieved, which was attributed to a significant improvement in the properties of the plasticized starch/polymer blend. The drastic reduction in tensile strength and elongation at break, usually observed in TPS polyester composite materials, does not occur for PA11 [36]. In turn, the elongation at break of the TPS/PA12 composite materials containing 30% wheat starch and produced using diglycidyl ether Bisphenol A and poly(ethylene-co-butyl acrylate-co-maleic anhydride) composite material as interphase tension reducing agents, was even improved [23].

The samples produced with 50% TPS in the composite material were characterized as having very good elastic behavior, expressed as percentage elongation of the sample at break. They did not differ significantly (statistically) in their elongation at break from the molded specimens produced from the polyamide alone. Only the addition of TPS in the amount of 70% slightly decreased the value of this parameter, and the addition of 90% TPS decreased the elongation at break of the samples by as much as 75%, compared with the samples with an equal share of TPS and PA (Table 1). Landreau et al. (2009) [36] produced TPS/PA11 composite materials containing up to 70% TPS using carboxymethylcellulose (CMC) using a single-stage, and a two-stage extrusion process. The ultimate product was characterized as not only having a high tensile strength above 15 MPa, but also by very good results of elongation at break [36]. In the work of these authors, the elongation at break of the composite materials with 70% TPS, did not differ from that of the composite materials containing only 40% TPS. Such good mechanical properties of the TPS-rich composite materials are a compromise of several mutually canceling effects. On the one hand, reducing the PA11 content in the composite materials leads to a decrease in mechanical strength. On the other hand, the changes in the morphology of the composite material structure occur with an increase in the co-continuity of the phases, and an increase in the tensile properties through a better stress distribution within the sample; this is related to the presence of CMC [14]. Although Landreau did not analyze the influence of double extrusion on the elongation at break of these samples, it can be speculated that it was the additional effect of phase mixing that contributed to such excellent elongation at break results of the composite materials with a high TPS content. This fact may explain why there was no increase in the elongation at break in the samples rich in PA11, compared with the TPS70 at the same CMC level [36]. The results reported by Landreau were confirmed in our study. No CMC addition to the composite materials analyzed in our study clearly confirms the influence of the two-stage manufacturing process on the elastic behavior of the molded specimens. It is probable that the better dispersion of the TPS resulting from the re-extrusion process observed in the samples containing less PA, contributed to an improvement of the phase continuity and, thus, to the elongation at break. This effect is not observed in the samples with a high PA content in which the TPS dispersion was already high after the first extrusion process. This fact was also confirmed by [34], who observed agglomerates of starch granules in the SEM image.

In further studies, it seems necessary to determine the effect of adding various plasticizers on the properties of the material produced, using the two-stage method described here. It is likely that by using such a procedure, it will be possible to obtain an even better degree of dispersion of both phases. In future works, the authors of this study will focus on optimizing the compatibility of all the above conditions. All of the materials obtained will be divided into groups according to their specific possible applications.

## 4. Conclusions

The extrusion process used as a preliminary stage in TPS/PA12 composite material manufacture produces a material with a high (up to 90%) TPS content from which molds can then be formed. It is impossible to produce such molds from starch-polyamide blends that have only been subjected to press molding.

The addition of TPS to PA increases its susceptibility to water; however, this effect does not worsen with higher TPS content in the composite material. This is illustrated by the lack of differences in theoretical water absorption across all tested TPS/PA composite materials at both measurement temperatures. Similarly, in the case of the practical water absorption of the composite materials determined at 30 °C, no statistically significant differences were shown in its value depending on the proportion of the starch component in the composite materials.

The solubility of the preparations at 80 °C increased with an increase in the starch content of the material. In water at a temperature of 30 °C, the preparations with 90% starch content were also dissolved to the greatest extent, and their average solubility reached 19.26%. However, no statistically significant differences were found in the solubility of the specimens with less than 90% starch content. In these samples, the solubility was more than twice lower, compared with the composite materials produced with the highest starch content.

The results of the mechanical properties and from the photos of preparations produced using the one- and two-stage plastics forming processes, indirectly confirmed the achievement of better dispersion of the material obtained using the pressing process preceded by the extrusion process.

All the mechanical properties tested decreased inversely proportional to the starch content. The samples produced with 50% TPS in the composite material were characterized as having a high percentage elongation at break. They did not differ significantly (statistically) in their elongation at break from the molds produced from the polyamide alone. Only the addition of TPS in the amount of 70% slightly decreased the value of this parameter.

PA composite materials containing up to 70% TPS should find practical applications as biodegradable materials, with functional properties that are acceptable to consumers which do not differ significantly from those using polyamides.

## Figures and Tables

**Table 1:** Mechanical properties of composite material with different TPS content.

TPS Content	Force [mN]	Tensile Strength [mPa]	Work Necessary to Break [mJ]	Elongation of the Sample at Break [%]
0%	77.03 a	3.94 a	48.21 a	2.55 a
50%	68.37 b	3.42 b	39.43 b	2.13 a
70%	63.39 b	3.17 b	32.59 b	1.58 b
90%	53.59 c	2.68 c	9.66 c	0.60 c

a,b,c—different letters are statistically different at *p* < 0.05.

## Data Availability

The original contributions presented in the study are included in the article, further inquiries can be directed to the corresponding author.

## References

[1] Pilicode N., Naik P., Nimith K.M., Acharya M., Satyanarayan M.N., Adhikari A.V. (2021). New cyanopyridine-based π-conjugative poly(azomethine)s: Synthesis, characterization and electroluminescence studies. Polym. Adv. Technol..

[2] Sunitha M.S., Naik P., Vishnumurthy K.A., Adhikari A.V. (2025). Synthesis of nonlinear heteroaromatic donor–acceptor conjugated polymers: Structural, theoretical, electrochemical, and optical properties. Polym. Eng. Sci..

[3] Zarski A., Bajer K., Kapuśniak J. (2021). Review of the most important methods of improving the processing properties of starch toward non-food applications. Polymers.

[4] Fourati Y., Magnin A., Putaux J.-L., Boufi S. (2020). One-step processing of plasticized starch/cellulose nanofibrils nanocomposites via twin-screw extrusion of starch and cellulose fibers. Carbohydr. Polym..

[5] Hietala M., Mathew A.P., Oksman K. (2013). Bionanocomposites of thermoplastic starch and cellulose nanofibers manufactured using twin-screw extrusion. Eur. Polym. J..

[6] Piekarska K., Sikora M., Owczarek M., Jóźwik-Pruska J., Wiśniewska-Wrona M. (2023). Chitin and Chitosan as Polymers of the Future—Obtaining, Modification, Life Cycle Assessment and Main Directions of Application. Polymers.

[7] Hisham F., Akmal M.H.M., Ahmad F., Ahmad K., Samat N. (2024). Biopolymer chitosan: Potential sources, extraction methods, and emerging applications. Ain Shams Eng. J..

[8] Alix S., Mahieu A., Terrie C., Soulestin J., Garault E., Feuilloley M.G.J., Gattin R., Edon V., Ait-Younes T., Leblanc N. (2013). Active pseudo-multilayered films from polycaprolactone and starch based matrix for food-packaging applications. Eur. Polym. J..

[9] Temesgen S., Rennert M., Tesfaye T., Nase M. (2021). Review on Spinning of Biopolymer Fibers from Starch. Polymers.

[10] Shahbaz M., Naeem H., Nayik G.A., Dar A.H. (2024). Application of starch as an active ingredient for the fabrication of nanocomposite in food packaging. Starch Based Nanomaterials for Food Packaging. Perspectives and Future Prospectus.

[11] Ning W., Jiugao Y., Xiaofei M. (2008). Preparation and characterization of compatible thermoplastic dry starch/poly(lactic acid). Polym. Compos..

[12] Muller C.M.O., Pires A.T.N., Yamashita F. (2012). Characterization of thermoplastic starch/poly(lactic acid) blends obtained by extrusion and thermopressing. J. Braz. Chem..

[13] Raj A., Yousfi M., Prashantha K., Samuel C. (2024). Morphologies, Compatibilization and Properties of Immiscible PLA-Based Blends with Engineering Polymers: An Overview of Recent Works. Polymers.

[14] Schwach E., Averous L. (2004). Starch-based biodegradable blends: Morphology and interface properties. Polym. Int..

[15] Avella M., Errico M.E. (2000). Preparation of PHBV/starch blends by reactive blending and their characterization. J. Appl. Polym. Sci..

[16] Parulekar Y., Mohanty A.K. (2007). Extruded Biodegradable Cast Films from Polyhydroxyalkanoate and Thermoplastic Starch Blends: Fabrication and Characterization. Macromol. Mater. Eng..

[17] Averous L., Fauconnier N., Moro L., Fringant C. (2000). Blends of thermoplastic starch and polyesteramide: Processing and properties. J. Appl. Polym. Sci..

[18] Oneesha H.P., Gunawardene O.H.P., Gunathilake C., Amaraweera S.M., Fernando N.M.L., Wanninayaka D.B., Manamperi A., Kulatunga A.K., Rajapaksha S.M., Dassanayake R.S. (2021). Compatibilization of Starch/Synthetic Biodegradable Polymer Blends for Packaging Applications: A Review. J. Compos. Sci..

[19] Mikus Y., Alix S., Soulestin J., Lacrampe M.F., Krawczak P., Coqueret X., Dole P. (2014). Deformation mechanisms of plasticized starch materials. Carbohydr. Polym..

[20] Mortazavi S., Ghasemi I., Oromiehie A. (2013). Effect of phase inversion on the physical and mechanical properties of low density polyethylene/thermoplastic starch. Polym. Test..

[21] Baniasadi H., Madani Z., Mohan M., Vaara M., Lipponen S., Vapaavuori J., Seppälä J.V. (2023). Heat-Induced Actuator Fibers: Starch-Containing Biopolyamide Composites for Functional Textiles. ACS. Appl. Mater. Interfaces.

[22] Teyssandier F., Cassagnau P., Gérard J.F., Mignard N., Mélis F. (2012). Morphology and mechanical properties of PA12/plasticized starch blends prepared by high-shear extrusion. Mater. Chem. Phys..

[23] Teyssandier F., Cassagnau P., Gérard J.F., Mignard N. (2011). Reactive compatibilization of PA12/plasticized starch blends: Towards improved mechanical properties. Eur. Polym. J..

[24] Rodriguez-Gonzalez F.J., Ramsay B.A., Favis B.D. (2003). High Performance LDPE/Thermoplastic Starch Blends: A Sustainable Alternative to Pure Polyethylene. Polymer.

[25] Hamdan S., Hashim D.M.A., Ahmad M., Embong S. (2000). Compatibility studies of polypropylene (PP)-sago starch (SS) blends using DMTA. J. Polym. Res. Taiwan.

[26] Lu Y., Tighzert L., Berzin F., Rondot S. (2005). Innovative plasticized starch films modified with waterborne polyurethane from renewable resources. Carbohydr. Polym..

[27] Puttonen T., Salmi M., Partanen J. (2021). Mechanical properties and fracture characterization of additive manufacturing polyamide 12 after accelerated weathering. Polym. Test..

[28] (2012). Plastics—Determination of Tensile Properties Part 2: Test Conditions for Moulding and Extrusion Plastics.

[29] Tomaszewska-Ciosk E., Zdybel E., Lech K., Nemś A. (2019). Effect of ethanol on properties of extrudates enriched with high-fibre by-products. Int. J. Food Sci. Tech..

[30] Richter M., Augustat S., Schierbaum F. (1968). Ausgewählte Methoden der Stärkechemie: Isolierung, Charakterisierung und Analytik von Stärkepolysacchariden.

[31] Drożdż W., Kapelko-Żeberska M., Zięba T., Gryszkin A., Tomaszewska-Ciosk E., Sielczak U. (2025). Production of Starch Esters by Roasting Potato Starch with Unripe Apple Juice. Appl. Sci..

[32] Ghasemlou M., Daver F., Murdoch B.J., Ball A.S., Ivanova E.P., Adhikari B. (2022). Biodegradation of novel bioplastics made of starch, polyhydroxyurethanes and cellulose nanocrystals in soil environment. Sci. Total Environ..

[33] Nazrin A., Sapuan S.M., Zuhri M.Y.M., Tawakkal I.S.M.A., Ilyas R.A. (2021). Water barrier and mechanical properties of sugar palm crystalline nanocellulose reinforced thermoplastic sugar palm starch (TPS)/poly(lactic acid) (PLA) blend bionanocomposites. Nanotechnol. Rev..

[34] Nazrin A., Sapuan S.M., Zuhri M.Y.M. (2020). Mechanical, Physical and Thermal Properties of Sugar Palm Nanocellulose Reinforced Thermoplastic Starch (TPS)/Poly (Lactic Acid) (PLA) Blend Bionanocomposites. Polymers.

[35] Li X., Ma C., Shi T., Yang H. (2025). Robust, fluorine-free, bioinspired PU superhydrophobic composite coating based on modified ceramics nanoparticle: Preparation, characterization and mechanism. Prog. Org. Coat..

[36] Landreau E., Tighzert L., Bliard C., Berzin F., Lacoste C. (2009). Morphologies and properties of plasticized starch/polyamide compatibilized blends. Eur. Polym. J..

[37] Anwajler B., Zdybel E., Tomaszewska-Ciosk E. (2023). Innovative Polymer Composites with Natural Fillers Produced by Additive Manufacturing (3D Printing)—A Literature Review. Polymers.

[38] Masanabo M.A., Ray S.S., Emmambux M.N. (2022). Properties of thermoplastic maize starch-zein composite films prepared by extrusion process under alkaline conditions. Int. J. Biol. Macromol..

[39] Li X., Li Z., Dong L., Liu B., Wang H., Shi T., Yuan S., Zhang Y., Ma C. (2025). Study of microstructure evolution and fatigue crack extension properties of 42CrMo steel strengthened by induction hardening. J. Mater. Res. Technol..

